# Evidence of validity of the Brazilian version of the Clinical Decision Making in Nursing Scale

**DOI:** 10.1590/0034-7167-2023-0351

**Published:** 2025-10-03

**Authors:** Maria Willian Freires Lopes, Flávio Rebustini, Isabel Cristina Kowal Olm Cunha, Elizabeth Bernardino, Alexandre Pazetto Balsanelli

**Affiliations:** I Universidade Federal de São Paulo. São Paulo, São Paulo, Brazil; II Universidade de São Paulo, School of Arts, Sciences and Humanities. São Paulo, São Paulo, Brazil; III Universidade Federal do Paraná. Curitiba, Paraná, Brazil

**Keywords:** Nursing, Clinical Decision-Making, Validation Study, Education, Translating., Enfermería, Toma de Decisiones, Estudio de Validación, Educación, Traducción

## Abstract

**Objective:**

to assess evidence of content validity and response process of the Clinical Decision Making in Nursing Scale for Brazilian Portuguese.

**Method:**

a psychometric study of cross-cultural adaptation. The committee of judges was multidisciplinary and comprised of 14 participants, using the Content Validity Coefficient for linguistic equivalence and content. The response process was carried out in two rounds.

**Results:**

in linguistic equivalence, the components and items of the instrument reached a Content Validity Coefficient above 0.57. In terms of content validity, items that did not reach the Content Validity Coefficient of 0.66 were rewritten or removed.

**Conclusions:**

the instrument translated and adapted to the Brazilian version represents a reassembly of the original. In terms of content validity, item wording is technically weak, requiring new tests for validity.

## RESUMEN

**Objetivo:**

evaluar la evidencia de validez de contenido y proceso de respuesta de la Clinical Decision Making in Nursing Scale para el idioma portugués hablado en Brasil.

**Método:**

estudio psicométrico de la adaptación transcultural. El panel de jueces fue multidisciplinario y estuvo compuesto por 14 participantes, utilizando el Coeficiente de Validez de Contenido para la equivalencia lingüística y de contenido. El proceso de respuesta se llevó a cabo en dos rondas.

**Resultados:**

en equivalencia lingüística, los componentes e ítems del instrumento alcanzaron un Coeficiente de Validez de Contenido superior a 0,57. En cuanto a la validez de contenido, los ítems que no alcanzaron el Coeficiente de Validez de Contenido de 0,66 fueron reescritos o eliminados.

**Conclusiones:**

el instrumento traducido y adaptado a la versión brasileña representa un reensamblaje del original. En términos de validez de contenido, la redacción de los ítems es técnicamente débil, requiriendo nuevas pruebas para su validación.

## INTRODUCTION

Decision-making is a fundamental skill for nurses in clinical practice in various healthcare settings at any level of care^(1)^. Furthermore, it is among one of the skills to be developed during training according to the Brazilian National Curricular Guidelines, as this professional must be grounded in the ability to make decisions with efficiency and cost-effectiveness of human, material and technical resources, requiring skills and abilities to assess, systematize and decide on conduct based on scientific evidence^(2)^.

The concept of clinical decision making (CDM) was introduced in the literature in the 1980s and is defined as the application of distinct thought patterns and data analysis, in conjunction with the multidisciplinary team, which uses clinical judgment to establish care procedures for patients^(3)^. CDM by nurses is a complex process that requires knowledge in relevant aspects of nursing, and has been considered an area of current scientific research that involves quality of care and patient safety^(3-5)^.

In scientific literature, there are CDM instruments such as Personal Problem-Solving Inventory^(6)^, Grover’s 126-items Nursing Performance Instrument^(7)^, General Decision-Making Style Questionnaire^(8)^, Participation in Decision Activities Questionnaire^(9)^, Nursing Decision Making Instrument^(7)^, each assessing a nuance of CDM. However, the Clinical Decision Making in Nursing Scale (CDMNS)^(10)^ specifically assesses nurses’ self-perception of their knowledge, values, beliefs and previous experience. In addition to being an instrument used in American studies, it has also been translated and validated in European countries and the Middle East^(11-13)^.

CDMNS is unavailable for use among Brazilian nurses, which is the problem of this study. The importance of translating a CDM scale to the Brazilian context will allow for multicenter studies, in addition to guiding conduct in undergraduate and residency courses, and even in continuing education programs, in various healthcare settings.

## OBJECTIVE

To assess evidence of validity of the Brazilian version of CDMNS.

## METHODS

### Ethical aspects

The study was approved by the *Universidade Federal de São Paulo* Research Ethics Committee, respecting the ethical aspects of research involving human beings recommended by Resolutions 466/2012 and 510/2016 of the Brazilian National Health Council.

### Study design

This is a psychometric study of cross-cultural adaptation (CCA) and validity of CDMNS for Brazilian Portuguese. To guide the sequencing of CCA stages, the study followed the procedures in the Functional Assessment Cancer Therapy (FACT)^(14)^, which are: translation, reconciliation, review by the original authors of the instrument; review by a committee of judges; pre-finalization review; finalization; harmonization and quality assurance; formatting, composition and proof-reading; cognitive testing and linguistic validity; comment analysis; and CCA finalization, with the final version in Brazilian Portuguese being obtained.

### Population or sample

Professionals with training and experience in competencies and/or CDM, with knowledge of psychometric studies, translators, nurses and psychologists were invited to the judging committee; this selection was carried out through the *Lattes* Platform and snowball sampling. In the response process phase, the instrument was applied to a sample of five clinical nurses.

### Study protocol

The translation stage of the scale was carried out by two independent translators with semantic, conceptual and cultural proficiency in the language of the original instrument. A health translator (T1) and a lay translator (T2) were selected.

Due to this process, a reconciliation of the two versions obtained from the initial translation stage (ST1-2) was carried out by a third independent Brazilian translator with proficiency in the original language, producing a preliminary final version.

The two versions were analyzed by a third translator, to reconcile the content in case of discrepancies in initial translations. From this version (ST1-2), two independent back-translations were carried out, as we did not obtain a consensus on the original version by the original author.

After the synthesis of translation of the pre-final version, a form was created in Google Forms^®^ to be sent to the study’s judging committee. The form content consisted of an assessment of linguistic equivalence (conceptual, cultural, idiomatic, semantic) and content. Concerning content, questions were asked about the clarity, relevance, and pertinence of the entire content of the instrument, including directions for filling out the instrument and the response format. Moreover, we added the following questions to assess the instrument in general: does the instrument meet the latent variable (CDM)? Does the instrument measure ONLY what it was intended to measure? Do the items meet the postulated concept? Is the language adopted appropriate for the population? Are the proposed dimensions consistent with the items? Is the scale appropriate for measuring the instrument items? Does the instrument meet the proposed dimensions? For each item questioned, a judge should answer “NO (0)” and “YES (1)”, and in case of a negative answer, justifications and suggestions were requested.

In the response process stage, a characterization questionnaire was sent, directed by a link, for completion of CDMNS-Br, and the Quilgo add-on was added to Google Forms^®^ to account for response time. Then, a cognitive interview was conducted on the instrument general appearance and item reading ability and understanding.

### Study analysis and statistics

To calculate validity content, the Content Validity Ratio (CVR)^(15)^ and the Content Validity Coefficient were adopted for all criteria, such as linguistic equivalence, content and questioning about the overall assessment of the instrument. This calculation is based on the number of participating judges. The comments scored by the committee of judges were analyzed to identify discrepancies in linguistic equivalence in translations and content, in order to determine the final version.

## RESULTS

The judging committee was composed of 14 professionals: a journalist, a psychologist, a translator with a degree in languages and linguistics, and 11 nurses. It was observed that ten judges (71.4%) had experience in psychometric studies and 11 nurses (78%) worked in different areas of nursing, from direct patient care to teaching. A CVR of 0.57 was considered for linguistic equivalence, and a CVR of 0.67 for questions related to content. It is worth noting that the translator with a degree in languages and linguistics and the journalist participated in the instrument equivalence and semantic analysis stage, and were not asked to assess the instrument content. The psychologist remained in the content stage due to her experience with psychometric studies. Table 1 presents the instrument’s semantic, idiomatic, cultural, and conceptual equivalences.

**Table 1:** Equivalence analysis of the preliminary version of the translation of the Clinical Decision Making in Nursing Scale into Brazilian Portuguese. São Paulo, SP, Brazil, 2022

Preliminary scale	Equivalences			
	SEM	IDIO	CULT	CONC
*ESCALA DE JENKINS PARA TOMADA DE DECISÃO CLÍNICA EM ENFERMAGEM*	1.00	1.00	0.86	0.71
*Instruções: Para cada uma das seguintes afirmações, pense em suas ações ao cuidar de pacientes em ambiente de cuidados intensivos. Responda com base no que você está fazendo agora no contexto clínico.*	0.85	1.00	1.00	0.86
*Não há respostas certas ou erradas. O importante é sua avaliação de como você normalmente age como um tomador de decisões no ambiente clínico. Nenhuma das afirmações abrangem situações de emergência. Circule a resposta que mais se aproxima da maneira como você se comporta. Não é necessário ficar muito tempo em cada resposta.*	0.86	1.00	1.00	1.00
*Responda todos os itens. Isso deve levar aproximadamente dez minutos.*	1.00	1.00	1.00	1.00
*Por favor, utilize a seguinte legenda ao responder a estas perguntas:*	0.71	1.00	0.86	0.86
*Circule de acordo com a forma como você provavelmente se comportaria:*	0.86	1.00	0.86	0.86
*S - Sempre - O que você faz consistentemente todas as vezes*	1.00	0.86	1.00	1.00
*F - Frequentemente - O que você costuma fazer na maioria das vezes*	1.00	1.00	1.00	1.00
*O - Ocasionalmente - O que você faz de vez em quando*	1.00	1.00	1.00	1.00
*R - Raramente - O que faz você raramente*	1.00	1.00	0.86	1.00
*N - Nunca - O que você nunca faz em nenhum momento*	1.00	1.00	1.00	1.00
*Nota: Certifique-se de responder em termos do que está fazendo no ambiente clínico no presente momento*	0.86	0.86	0.71	0.71
*1 - Se a decisão clínica for vital e houver tempo, faço uma busca completa por alternativas.*	1.00	1.00	1.00	1.00
*2 - Quando uma pessoa está doente, seus valores culturais e crenças são secundários à implementação dos serviços de saúde.*	1.00	1.00	1.00	1.00
*3 - Os fatores situacionais do momento determinam o número de opções que exploro antes de tomar uma decisão.*	1.00	1.00	1.00	1.00
*4 - A busca por novas informações ao tomar uma decisão traz mais problemas que compensações.*	1.00	1.00	1.00	1.00
*5 - Eu consulto livros ou literatura profissional para pesquisar coisas que não entendo.*	1.00	1.00	0.86	1.00
*6 - Uma abordagem aleatória para procuraras opções funciona melhor para mim.*	0.71	0.71	1.00	1.00
*7 - Eu utilizo do método Brainstorming para encontrar ideias pra soluções*	1.00	0.71	0.71	1.00
*8 - Eu me empenho para obter o máximo de informações possível para tomar decisões.*	1.00	1.00	1.00	1.00
*9 - Eu ajudo meus pacientes no exercício de seus direitos de tomar decisões sobre seus próprios cuidados*	0.86	0.86	1.00	1.00
*10 - Quando meus valores estão em conflito com os do cliente, sou objetivo o suficiente para lidar com a tomada de decisão necessária para a situação.*	0.86	1.00	0.86	1.00
*11 - Eu considero os conselhos e opiniões de especialistas, mesmo que essa não seja a escolha que eu faria.*	0.71	1.00	0.86	1.00
*12 - Eu resolvo um problema ou tomo uma decisão sem consultar ninguém, utilizando as informações de que disponho no momento.*	1.00	1.00	1.00	1.00
*13 - Nem sempre paro para examinar todas as possíveis consequências de uma decisão que preciso tomar.*	0.86	0.86	1.00	1.00
*14 - Considero o bem-estar futuro da família quando tomo uma decisão clínica que envolve o paciente.*	1.00	1.00	0.86	1.00
*15 - Tenho pouco tempo ou energia disponível para buscar informações.*	1.00	1.00	1.00	1.00
*16 - Eu listo mentalmente as opções antes de tomar uma decisão.*	0.86	1.00	1.00	1.00
*17 - Ao examinar as consequências das opções que posso escolher, geralmente penso “Se eu fizer isso, então ...”.*	1.00	1.00	1.00	1.00
*18 - Eu considero até as consequências mais remotas antes de fazer uma escolha.*	0.86	1.00	1.00	1.00
*19 - O consenso entre meus pares é importante para mim para a tomada de decisão.*	0.86	1.00	1.00	1.00
*20 - Eu incluo os pacientes como fonte de informações*	0.86	1.00	1.00	1.00
*21 - Eu considero o que meus pares vão dizer quando penso nas possíveis escolhas que eu poderia fazer.*	0.86	1.00	0.86	1.00
*22 - Se um profissional mais experiente recomenda uma opção em uma situação de tomada de decisão clínica, eu a adoto em vez de procurar outras opções.*	0.71	0.86	0.71	0.86
*23 - Se um benefício for realmente grande, vou favorecê-lo sem olhar para todos os riscos.*	1.00	1.00	1.00	1.00
*24 - Eu procuro por novas informações aleatoriamente.*	0.86	1.00	1.00	1.00
*25 - Minhas experiências anteriores têm pouca relação com a forma como observo ativamente os riscos e benefícios em decisões sobre os pacientes.*	0.86	1.00	1.00	0.86
*26 -Ao examinar as consequências das opções que posso escolher, estou ciente dos resultados positivos para o meu paciente.*	0.85	1.00	1.00	1.00
*27 - Eu seleciono opções que obtive sucesso em circunstâncias semelhantes no passado.*	0.86	1.00	1.00	1.00
*28 - Se os riscos forem sérios o suficiente para causar problemas, descarto a opção.*	1.00	1.00	1.00	1.00
*29 - Eu escrevo uma lista de consequências positivas e negativas quando estou avaliando uma decisão clínica importante.*	0.86	1.00	1.00	0.86
*30 - Não peço aos meus pares que sugiram opções para minhas decisões clínicas.*	1.00	1.00	0.86	0.86
*31 - Meus valores profissionais são inconsistentes com meus valores pessoais.*	1.00	1.00	1.00	1.00
*32 - Para mim encontrar alternativas parece ser em grande parte uma questão de sorte.*	0.67	0.83	0.83	1.00
*33 - No ambiente clínico, tenho em mente os objetivos para a rotina do dia.*	1.00	1.00	1.00	1.00
*34 - Os riscos e benefícios são as coisas mais distantes da minha mente quando tenho que tomar uma decisão.*	0.86	1.00	1.00	0.86
*35 - Quando tenho que tomar uma decisão clínica, considero as prioridades e padrões institucionais*	0.86	0.86	0.86	0.71
*36 - Eu envolvo outras pessoas na minha tomada de decisão somente se a situação exigir.*	0.86	1.00	1.00	1.00
*37 - Em minha busca por opções, incluo até mesmo aquelas que podem ser consideradas “absurdas” ou inviáveis.*	1.00	1.00	1.00	0.86
*38 - Saber mais sobre os objetivos do paciente faz parte da minha tomada de decisão clínica.*	0.86	1.00	1.00	1.00
*39 - Examino os riscos e benefícios apenas para consequências que tenham implicações sérias.*	1.00	1.00	1.00	0.86
*40 - Os valores dos pacientes devem ser consistentes com os meus para que eu possa tomar uma boa decisão.*	1.00	1.00	1.00	1.00

*Note: SEM - semantic; IDIO - idiomatic; CULT - cultural; CONC - conceptual.*

It is noted that all components and items of the adapted instrument achieved a CVR score above 0.57 in linguistic equivalence.

Table 2 shows the Content Validity Coefficient of the preliminary version of CDMNS regarding pertinence, clarity and relevance. Moreover, items 6 and 32 were excluded, as they did not score the expected CVR of 0.67 in the relevance domain. Items 2, 19 and 37, with a score of 0.67 in the relevance and clarity domain, were revised and rewritten according to judges’ suggestions.

**Table 2:** Clinical Decision Making in Nursing Scale Content Validity Coefficient, preliminary version. São Paulo, SP, Brazil, 2022

Preliminary scale	Pertinence	Clarity	Relevance
THE JENKINS’ CLINICAL DECISION MAKING IN NURSING SCALE	1.00	1.00	1.00
Directions: For each of the following statements, think of your behavior while caring for clients in the critical care environment. Answer on the basis of what you are doing right now in the clinical setting.	1.00	0.83	1.00
There are no “right” or “wrong” answers. What is important is your assessment of how you ordinarily operate as a decision maker in the clinical setting. None of the statements cover emergency situations. Circle the answer that comes closest to the way you behave. Do not dwell on the responses.	0.83	1.00	1.00
Answer all items. This should take appropriately ten minutes.	1.00	1.00	1.00
Please use the following scale when answering these questions:	1.00	1.00	1.00
Circle whether you would likely behave in the described way:	1.00	1.00	1.00
A - Always - What you consistently do every time	1.00	1.00	1.00
F - Frequently - What you usually do most of the time	1.00	0.83	1.00
O - Occasionally What you sometimes do on occasion	1.00	1.00	1.00
S - Seldom - What you rarely do	1.00	0.83	1.00
N - Never - What you never do at any time	1.00	0.83	1.00
Note: Be sure you respond in terms of what you are doing in the clinical setting at the present time.	**0.67**	0.83	1.00
1 - If the clinical decision is vital and there is time, I conduct a thorough search for alternatives.	1.00	**0.67**	1.00
2 - When a person is ill, his or her cultural values and beliefs are secondary to the implementation of health services.	1.00	**0.67**	1.00
3 - The situational factors at the time determine the number of options that I explore before making a decision.	0.83	1.00	1.00
4 - Looking for new information in making a decision is more trouble than it’s worth.	1.00	1.00	1.00
5 - I use books or professional literature to look up things I don’t understand.	1.00	0.83	1.00
6 - A random approach for looking at options works best for me.	**0.50**	**0.50**	1.00
7 - Brainstorming is a method I use when thinking of ideas for options.	1.00	1.00	1.00
8 - I go out of my way to get as much information as possible to make decisions.	1.00	0.83	1.00
9 - I assist clients in exercising their rights to make decisions about their own care.	1.00	1.00	1.00
10 - When my values conflict with those of the client, I am objective enough to handle the decision making required for the situation	1.00	1.00	1.00
11 - I listen to or consider expert advice or judgment, even though it may not be the choice I would make.	1.00	1.00	1.00
12 - I solve a problem or make a decision without consulting anyone, using information available to me at the time.	1.00	1.00	1.00
13 - I don’t always take time to examine all the possible consequences of a decision I must make.	0.83	1.00	1.00
14 - I consider the future welfare of the family when I make a clinical decision which involves the individual.	1.00	1.00	1.00
15 - I have little time or energy available to search for information.	1.00	**0.67**	1.00
16 - I mentally list options before making a decision.	1.00	1.00	1.00
17 - When examining consequences of options I might choose, I generally think through “If I did this, then. . .”.	1.00	1.00	1.00
18 - I consider even the remotest consequences before making a choice.	1.00	1.00	1.00
19 - Consensus among my peer group is important to me in making a decision.	1.00	0.83	1.00
20 - I include clients as sources of information.	1.00	1.00	1.00
21 - I consider what my peers will say when I think about possible choices I could make.	1.00	1.00	1.00
22 - If an instructor recommends an option to a clinical decision-making situation, I adopt it rather than searching for other options.	1.00	1.00	1.00
23 - If a benefit is really great, I will favor it without looking at all the risks.	1.00	1.00	1.00
24 - I search for new information randomly.	1.00	1.00	1.00
25 - My past experiences have little to do with how actively I look at risks and benefits for decisions about clients.	1.00	**0.67**	1.00
26 - When examining consequences of options I might choose, I am aware of the positive outcomes for my client.	1.00	1.00	1.00
27 - I select options that I have used successfully in similar circumstances in the past.	1.00	1.00	1.00
28 - If the risks are serious enough to cause problems, I reject the option.	1.00	1.00	1.00
29 - I write out a list of positive and negative consequences when I am evaluating an important clinical decision.	1.00	**0.67**	1.00
30 - I do not ask my peers to suggest options for my clinical decisions.	1.00	1.00	1.00
31 - My professional values are inconsistent with my personal values.	1.00	1.00	1.00
32 - My finding of alternatives seems to be largely a matter of luck.	**0.50**	1.00	1.00
33 - In the clinical setting I keep in mind the course objectives	1.00	1.00	1.00
34 - The risks and benefits are the farthest thing from my mind when I have to make a decision	1.00	**0.50**	1.00
35 - When I have a clinical decision to make, I consider the institutional priorities and standards.	1.00	1.00	1.00
36 - I involve others in my decision making only if the situation calls for it.	1.00	1.00	1.00
37 - In my search for options, I include even those that might be thought of as “far out” or not feasible.	1.00	0.83	1.00
38 - Finding out about the client’s objectives is a regular part of my clinical decision making.	1.00	0.83	1.00
39 - I examine the risks and benefits only for consequences that have serious implications	1.00	**0.67**	1.00
40 - The client’s values have to be consistent with my own in order for me to make a good decision.	1.00	0.83	1.00

From the technical point of view of item writing, there were items with two factors (1, 2, 12, 15, 25, 34, 37 and 39) and the need to adapt items (3, 5, 9, 11, 13, 28, 29, 35). It is noted that, after adjustments of these technical issues, an instrument with 24 items that needed modification emerged, with eight items having two factors and 16 items being rewritten. Thus, we can note that these modifications configure an assembly^(16)^.

Table 3 presents the analysis of the instrument in general, considering a CVR of 0.67 for each question.

**Table 3:** Validity coefficient of the preliminary version of the Clinical Decision Making in Nursing Scale. São Paulo, SP, Brazil, 2022

Questions on the instrument	CVR
Does the instrument measure ONLY what it was intended to measure?	**0.50**
Do the items meet the postulated concept?	0.67
Is the language used appropriate for the population?	0.67
Are the proposed dimensions consistent with the items?	**0.45**
Is the scale appropriate for measuring the instrument items?	**0.37**
Does the instrument meet the proposed dimensions?	**0.50**

*Note: CVR - Content Validity Ratio.*

It is noted that in the proposed dimensionality scale, latent variable studied, in the case of CDM, CVR was below expectations.

In the response process phase, it was composed of ten nurses with an average age of 28 years, six of whom were female and had completed five years of training (SD 1.18). Furthermore, eight had a specialization in the residency modality; seven worked in an Intensive Care Unit; and three worked in an inpatient unit. The length of service at the institution was 2.2 years (SD 1.32), and the length of service in the current sector was 1.7 years (SD 0.9). This stage was carried out in two rounds. In the first round, the preliminary version of the instrument was applied to five nurses recruited from the target population. All comments and suggestions for each item were compiled and analyzed. Participants did not report any difficulty in reading the instrument or filling out directions. It was observed that all items were answered, and the dismembered items were arranged in a different order, to prevent participants from being confused while filling out the instrument. In this first round, the item “*Escrevo uma lista de consequências, quando estou avaliando uma decisão clínica importante*” was modified, because the act of writing was considered an activity that is not done, replacing it with “*Listo mentalmente as consequências quando estou avaliando uma decisão clínica importante*”. Item 37 was considered to be outside the scope of the action, in addition to putting patients at risk. Given the findings, a second round was held with five nurses; in this phase, no difficulties were reported by participants. It was observed that the average response time was ten minutes (minimum time of seven to 13 minutes).

## DISCUSSION

CDM is a nursing task that requires technical and scientific knowledge. A CDM instrument will contribute to improving university curricula, supporting the difficulties faced by undergraduate nursing students. Hospitals will be able to develop their professionals to have self-efficacy in the decision-making process in their work settings and thus be able to obtain better care outcomes.

Choosing a CDM instrument developed in another culture and language requires CCA. This is a complex process that involves a sequence of methodological procedures to ensure that the latent variable is being measured, despite a different context. Furthermore, this does not guarantee that we will have an instrument adapted and validated in our culture and population, even following the contemporary psychometrics recommendations^(16,17)^.

It is important to assess linguistic equivalence guided by the cultural context of the latent variable studied for Brazilian nurses. From this perspective, the cultural context of Brazilian nurses’ CDM competency in the current scenario is noteworthy, since the instrument was developed in the 1980s, in American nurses’ historical and cultural context. CDMNS is the most widely used instrument in scientific publications in several countries around the world regarding CDM research.

In the present study, two methodological frameworks were used with the FACT protocol recommendations^(14)^, aligned with the Beaton *et al*. framework^(18)^, providing greater robustness in this process. There were no significant discrepancies in the versions presented by the translators, and those that did arise were resolved by the researchers. It was observed that achieving equivalence is not enough, as it would replicate possible errors in the instrument; therefore, it is necessary to hold a committee of judges to verify other issues related to content and the instrument in general^(16)^.

However, only in Kouravand’s work^(19)^, who adapted and validated CDMNS for Persian culture, the Beaton protocol was adopted for CCA sequencing. Other articles did not mention guiding protocols, contributing to fragility^(20-22)^.

Within CCA process, in the Brazilian version, back-translation was not performed, due to the public domain of the instrument after the author’s death. This stage has been reported by Epstein *et al*.^(23)^ as having a moderate impact. The committee of judges, in turn, helps to ensure accurate content, considering that they are bilingual and knowledgeable about the latent variable in the context to be adapted. One of the main functions of the committee is to ensure that the questionnaire content remains true to the original.

Another important point was the adoption of CVR applied to the analysis of judges’ responses, using dichotomous score, which requires judges’ stance regarding their choices; this index is superior to the Content Validity Index and allows the inclusion of a greater number of judges^(24,25)^. This is contrary to the Polit Content Validity Index recommendation ^(26)^, which can usually generate bias, since the index is not adjusted to the number of judges as in the critical CVR. However, Epstein *et al*.^(23)^ recommendations specify that the committee of judges should be composed of researchers, translators, and methodological and lay professionals. Given these more recent recommendations, it would be risky to restrict the opinions to five experts, which could bias the analyses.

From the results obtained, this study differed from publications on CDMNS validity in other countries^(20-22)^, since the adapted translation presents technical issues in the wording of the instrument scored by the committee of judges, such as items with two factors, weaknesses in the scale format and suggestions for reformulating wording. These results allow us to say that CCA process is classified as a reassembly, since the reformulation of the adapted content diverges from the original^(16)^.

Considering the above, Coons^(27)^ and Steward^(28)^ state that equivalence rigor should vary according to the magnitude of the modifications. However, regardless of the level of modifications, it is recommended to test the modifications through cognitive interviews, usability testing and psychometric measures^(25)^.

At the heart of the changes to the instrument’s response format, in the study by Minish of Palestine^(22)^, the committee of judges suggested the use of a 3-point Likert-type scale (1 = never; 2 = frequently; 3 = occasionally). A study by Girot^(29)^ uses a 5-point Likert-type scale of agreement, in accordance with the first publication on the development of the instrument^(10)^. To test these modifications, usability testing and general psychometric properties are suggested.

In this context, the development of an instrument, even if adapted, requires good writing and an appropriate scale format to measure the latent variable, which will minimize the impact of the internal structure and provide interpretation of results for both researchers and users of the instrument^(17)^.

In validity studies of CDMNS by Duarte^(30)^ and Kouravand^(19)^, 17 items were excluded from the original instrument in the internal structure phase. In this study, it was noted that, in the test content phase, some of the items mentioned had already been excluded. The relevance of the characteristic of the multidisciplinary committee of judges, with experience in psychometric studies, stood out, which, in addition to analyzing the instrument content, also verifies other aspects related to item wording, response format and the understanding of items in the response process stage.

There was the need to reassemble CDMNS in the Brazilian version. According to Iliescu, the reassembly of an instrument concerns a new measurement in the target culture, where new content was generated. Given the result of the modifications to the items, new content was generated that requires reflection on CDM in the Brazilian context and the dilemma of nurses’ self-perception in their practice, which may be an option to be developed to provide the Brazilian culture with a functional, useful and valid measurement of the target construct.

Another important point about importing the instrument into Brazilian culture, as mentioned previously, refers to the issue of dealing with a continental country with its socioeconomic-cultural differences and hospital management models, which can be considered as important contextual factors when considering the original instrument’s cultural context.

It is noted that the published CDMNS validity articles^(20-22)^ refer to the response process as pre-test, without giving its correct nomenclature, and little is reported about how the techniques are performed, i.e., it is mentioned how the pre-test was performed, although response time is not taken into account^(20-22)^. In the original article^(10)^, response time was similar to that presented in this study, which was 12 minutes.

### Limitations of the Study

This study has some limitations. CDMNS, translated into Brazilian Portuguese, is a material developed in previous decades that, today, can have a completely different impact when applied to the context, given the generational changes of nurses and the diversity of hospital characteristics in the scenario of our continental country. In the adaptation process, it must be ensured that the instrument’s concept is the same as the original not only for language, but also for time and context. It is possible to consider the barriers when making a direct comparison between nations, cultures and time. It was necessary to have two committees of judges to assess equivalence and another committee of judges to assess content that covers the regions of our continental country.

### Contributions to health, nursing or public policies

The findings of this study contribute to the advancement of knowledge in nursing, helping to improve the weaknesses found regarding CDM at the bedside, both for students and for nurses. It is important other studies with contemporary framework that guide the process of reassembling the instrument for the Brazilian context, continuing the process of evidence of validity of a CDM instrument for nurses in our country.

## CONCLUSION

CDMNS was translated and culturally adapted for Brazil according to the methodology recommended in literature. The Brazilian version represents a reassembly of the original instrument, demonstrating linguistic equivalence, but it will need to be tested in its internal structure to verify interpretation of items. Evidence of test content is technically weak, requiring tests for its validity. The response process presents good evidence of validity in item content interpretation by participants. This instrument will serve as a strategy to provide subsidies to universities and organizations, promoting strategies in nursing curricula and to contributing to the continuous development in organizations to support nurses’ CDM in their work settings.

## Data Availability

Research data is available in the body of the article.
