# Supplementary material for: Evidence of validity of the Brazilian version of the Clinical Decision Making in Nursing Scale
Source: Rev Bras Enferm. 2025 Oct 3;78(4):e20230351. doi: 10.1590/0034-7167-2023-0351 (PMC12494353; doi:10.1590/0034-7167-2023-0351)
Supplement: Translation

# Translation: Evidências de validade do instrumento *Clinical Decision Making in Nursing Scale* na versão brasileira

**DOI:** 10.1590/0034-7167-2023-0351pt

**Authors:** Maria Willian Freires Lopes, Flávio Rebustini, Isabel Cristina Kowal Olm Cunha, Elizabeth Bernardino, Alexandre Pazetto Balsanelli

**Affiliations:** I Universidade Federal de São Paulo. São Paulo, São Paulo, Brasil; II Universidade de São Paulo, Escola de Artes, Ciências e Humanidades. São Paulo, São Paulo, Brasil; III Universidade Federal do Paraná. Curitiba, Paraná, Brasil

**Keywords:** Enfermagem, Tomada de Decisão Clínica, Estudos de Validação, Educação, Processo de Tradução

## Abstract

**Objetivo:**

avaliar as evidências de validade de conteúdo e processo de resposta do instrumento *Clinical Decision Making in Nursing Scale* para o português falado no Brasil.

**Método:**

estudo psicométrico de adaptação transcultural. O painel de juízes foi multidisciplinar composto por 14 participantes, utilizando-se o Coeficiente de Validade de Conteúdo para equivalência linguística e conteúdo. O processo de resposta foi realizado em duas rodadas.

**Resultados:**

na equivalência linguística, os componentes e itens do instrumento atingiram o Coeficiente de Validade de Conteúdo acima de 0,57. Na validade de conteúdo, os itens que não atingiram o Coeficiente de Validade de Conteúdo de 0,66 foram reescritos ou retirados.

**Conclusões:**

o instrumento traduzido e adaptado para a versão brasileira representa uma remontagem do original. Na validade de conteúdo, a redação dos itens apresenta fragilidade do ponto de vista técnico, com necessidade de novos testes para a sua validação.

## INTRODUÇÃO

A tomada de decisão é uma competência fundamental para o enfermeiro na prática clínica em diversos cenários de assistência à saúde de qualquer nível de atenção^(1)^. Além disso, está entre uma das competências a serem desenvolvidas durante a formação pelas Diretrizes Curriculares Nacionais, pois esse profissional deve estar fundamentado na capacidade de tomar decisões com eficácia e custo-efetividade de recursos humanos, material e técnico, exigindo competências e habilidades para avaliar, sistematizar e decidir sobre condutas baseadas em evidências científicas^(2)^.

O conceito de tomada de decisão clínica (TDC) foi introduzido na década de 1980 na literatura, e possui como definição a aplicação de padrões de pensamentos distintos e análise de dados, em conjunto com a equipe multiprofissional, que utiliza o julgamento clínico para estabelecer condutas de assistência prestada ao paciente^(3)^. A TDC pelo enfermeiro é um processo complexo que exige conhecimentos em aspectos relevantes em enfermagem, e tem sido considerada uma área de investigação científica atual que envolve a qualidade do cuidado e a segurança do paciente^(3-5)^.

Na literatura científica, há instrumentos de TDC como *Personal Problem-Solving Inventory* ^(6)^, *Grover’s 126-items Nursing Performance Instrument* ^(7)^, *General Decision-Making Style Questionnaire* ^(8)^, *Participation in Decision Activities Questionnaire* ^(9)^, *Nursing Decision Making Instrument^(^* ^7)^, cada um avaliando uma nuance da TDC . No entanto, o *Clinical Decision Making in Nursing Scale* (CDMNS)^(10)^ avalia especificamente a autopercepção do enfermeiro com relação aos seus conhecimentos, valores, crenças e experiência prévia. Além de ser um instrumento utilizado em estudos americanos, também foi traduzido e validado em países europeus e no Oriente Médio^(11-13)^.

O CDMNS está indisponível para o uso entre os enfermeiros brasileiros, sendo esse o problema da presente pesquisa. A importância da tradução de uma escala de TDC para o contexto brasileiro permitirá realizar estudos multicêntricos, além de nortear condutas nos cursos de graduação e de residências, e até mesmo em programas de educação permanente, em diversos cenários de assistência à saúde.

## OBJETIVO

Avaliar as evidências de validade do instrumento CDMNS na versão brasileira.

## MÉTODOS

### Aspectos éticos

O estudo foi aprovado pelo Comitê de Ética em Pesquisa do da Universidade Federal de São Paulo, respeitando-se os aspectos éticos de pesquisas envolvendo seres humanos preconizados pela Resoluções n^o^ 466/2012 e n^o^ 510/2016 do Conselho Nacional de Saúde.

### Desenho do estudo

Trata-se de estudo psicométrico de adaptação transcultural (ATC) e validação do CDMNS para a língua portuguesa falada no Brasil. Para nortear o sequenciamento das etapas de ATC, foram seguidos os procedimentos seguidos no *Functional Assessment Cancer Therapy* (FACT)^(14)^, sendo elas: tradução, reconciliação, revisão dos autores originais do instrumento; revisão do comitê de juízes; revisão pré-finalização; finalização; harmonização e garantia de qualidade; formatação, composição e prova de leitura; testagem cognitiva e validação linguística; análise dos comentários; e finalização da ATC, com a obtenção final da versão final em português falado no Brasil.

### População ou amostra

Para o comitê de juízes, foram convidados profissionais com formação e experiência em competências e/ou TDC, com conhecimentos em estudos psicométricos, tradutores, enfermeiros e psicólogos; esta seleção foi realizada por meio da Plataforma *Lattes* e por método bola de neve. Na fase de processo de resposta, o instrumento foi aplicado em uma amostra de cinco enfermeiros assistenciais.

### Protocolo de estudo

A etapa de tradução da escala foi realizada por dois tradutores independentes com domínio semântico, conceitual e cultural do idioma do instrumento original. Foram selecionados um tradutor da área da saúde (T1) e um tradutor leigo (T2).

Devido a esse processo, foi realizada uma reconciliação das duas versões obtidas da etapa inicial da tradução (ST1-2), realizada por um terceiro tradutor independente brasileiro com domínio do idioma original, produzindo uma versão final prévia.

As duas versões foram analisadas por um terceiro tradutor, para conciliar o conteúdo em caso de discrepâncias das traduções iniciais. A partir desta versão (ST1-2), foram realizadas duas retrotraduções independentes, como não obtivemos um consenso da versão original da autora original.

Após a síntese da tradução da versão pré-final, foi construído um formulário no *Google Forms^®^* para ser encaminhado ao comitê de juízes do estudo. O conteúdo do formulário consistiu na avaliação da equivalência linguística (conceitual, cultural, idiomática, semântica) e de conteúdo. Quanto ao conteúdo, foi questionado sobre a clareza, relevância e pertinência de todo o conteúdo do instrumento, incluindo as orientações de preenchimento e formato de resposta do instrumento. Além disso, acrescentamos as seguintes questões para avaliação do instrumento no geral: o instrumento atende à variável latente (TDC)? O instrumento mede APENAS o que se propôs? Os itens atendem ao conceito postulado? A linguagem adotada é adequada para a população? As dimensões propostas são coerentes com os itens? A escala é adequada para a mensuração dos itens do instrumento? O instrumento atende às dimensionalidades propostas? Para cada item questionado, o juiz deveria responder “NÃO (0)” e “SIM (1)”, e em caso de uma resposta negativa, foram solicitadas justificativas e sugestões.

Na etapa de processo de resposta, foi encaminhado um questionário de caracterização, direcionado por um *link*, para o preenchimento do CDMNS-Br, sendo adicionado o complemento *Quilgo* no *Google Forms^®^* para contabilizar o tempo de resposta. Em seguida, foi realizada uma entrevista cognitiva sobre a aparência geral do instrumento, capacidade de leitura e compreensão dos itens.

### Análise do estudo e estatística

Para o cálculo do conteúdo de validade, foram adotados o *Content Validity Ratio* (CVR)^(15)^ e o Coeficiente de Validade de Conteúdo para todos os critérios, como equivalência linguística, conteúdo e questionamento sobre avaliação geral do instrumento. Esse cálculo baseia-se no número de juízes participantes. Os comentários pontuados pelo comitê de juízes foram analisados para identificar as discrepâncias de equivalência linguística nas traduções e no conteúdo, para, assim, determinar a versão final.

## RESULTADOS

O comitê de juízes foi composto por 14 profissionais: um jornalista, um psicólogo, uma tradutora com formação em letras e 11 enfermeiros. Observa-se que dez dos juízes (71,4%) possuíam experiência em estudos psicométricos e 11 enfermeiros (78%) atuam em diferentes áreas da enfermagem, desde a assistência direta ao paciente à docência. Foram considerados um CVR de 0,57, para a equivalência linguística, e um CVR de 0,67, para as questões relacionadas ao conteúdo. Ressalta-se que a letróloga e o jornalista participaram da etapa de análise de equivalências e da análise semântica do instrumento, não sendo solicitada sua avaliação no conteúdo do instrumento. A psicóloga permaneceu na fase do conteúdo pelo critério de experiência com estudos psicométricos. A Tabela 1 apresenta as equivalências semântica, idiomática, cultural e conceitual do instrumento.

**Tabela 1:** Análise de equivalências da versão preliminar da tradução do *Clinical Decision Making in Nursing Scale* para o português falado no Brasil. São Paulo, SP, Brasil, 2022

Escala preliminar	Equivalências			
	SEM	IDIO	CULT	CONC
ESCALA DE JENKINS PARA TOMADA DE DECISÃO CLÍNICA EM ENFERMAGEM	1,00	1,00	0,86	0,71
Instruções: Para cada uma das seguintes afirmações, pense em suas ações ao cuidar de pacientes em ambiente de cuidados intensivos. Responda com base no que você está fazendo agora no contexto clínico.	0,85	1,00	1,00	0,86
Não há respostas certas ou erradas. O importante é sua avaliação de como você normalmente age como um tomador de decisões no ambiente clínico. Nenhuma das afirmações abrangem situações de emergência. Circule a resposta que mais se aproxima da maneira como você se comporta. Não é necessário ficar muito tempo em cada resposta.	0,86	1,00	1,00	1,00
Responda todos os itens. Isso deve levar aproximadamente dez minutos.	1,00	1,00	1,00	1,00
Por favor, utilize a seguinte legenda ao responder a estas perguntas:	0,71	1,00	0,86	0,86
Circule de acordo com a forma como você provavelmente se comportaria:	0,86	1,00	0,86	0,86
S - Sempre - O que você faz consistentemente todas as vezes	1,00	0,86	1,00	1,00
F - Frequentemente - O que você costuma fazer na maioria das vezes	1,00	1,00	1,00	1,00
O - Ocasionalmente - O que você faz de vez em quando	1,00	1,00	1,00	1,00
R - Raramente - O que faz você raramente	1,00	1,00	0,86	1,00
N - Nunca - O que você nunca faz em nenhum momento	1,00	1,00	1,00	1,00
Nota: Certifique-se de responder em termos do que está fazendo no ambiente clínico no presente momento	0,86	0,86	0,71	0,71
1 - Se a decisão clínica for vital e houver tempo, faço uma busca completa por alternativas.	1,00	1,00	1,00	1,00
2 - Quando uma pessoa está doente, seus valores culturais e crenças são secundários à implementação dos serviços de saúde.	1,00	1,00	1,00	1,00
3 - Os fatores situacionais do momento determinam o número de opções que exploro antes de tomar uma decisão.	1,00	1,00	1,00	1,00
4 - A busca por novas informações ao tomar uma decisão traz mais problemas que compensações.	1,00	1,00	1,00	1,00
5 - Eu consulto livros ou literatura profissional para pesquisar coisas que não entendo.	1,00	1,00	0,86	1,00
6 - Uma abordagem aleatória para procuraras opções funciona melhor para mim.	0,71	0,71	1,00	1,00
7 - Eu utilizo do método Brainstorming para encontrar ideias pra soluções	1,00	0,71	0,71	1,00
8 - Eu me empenho para obter o máximo de informações possível para tomar decisões.	1,00	1,00	1,00	1,00
9 - Eu ajudo meus pacientes no exercício de seus direitos de tomar decisões sobre seus próprios cuidados	0,86	0,86	1,00	1,00
10 - Quando meus valores estão em conflito com os do cliente, sou objetivo o suficiente para lidar com a tomada de decisão necessária para a situação.	0,86	1,00	0,86	1,00
11 - Eu considero os conselhos e opiniões de especialistas, mesmo que essa não seja a escolha que eu faria.	0,71	1,00	0,86	1,00
12 - Eu resolvo um problema ou tomo uma decisão sem consultar ninguém, utilizando as informações de que disponho no momento.	1,00	1,00	1,00	1,00
13 - Nem sempre paro para examinar todas as possíveis consequências de uma decisão que preciso tomar.	0,86	0,86	1,00	1,00
14 - Considero o bem-estar futuro da família quando tomo uma decisão clínica que envolve o paciente.	1,00	1,00	0,86	1,00
15 - Tenho pouco tempo ou energia disponível para buscar informações.	1,00	1,00	1,00	1,00
16 - Eu listo mentalmente as opções antes de tomar uma decisão.	0,86	1,00	1,00	1,00
17 - Ao examinar as consequências das opções que posso escolher, geralmente penso “Se eu fizer isso, então ...”.	1,00	1,00	1,00	1,00
18 - Eu considero até as consequências mais remotas antes de fazer uma escolha.	0,86	1,00	1,00	1,00
19 - O consenso entre meus pares é importante para mim para a tomada de decisão.	0,86	1,00	1,00	1,00
20 - Eu incluo os pacientes como fonte de informações	0,86	1,00	1,00	1,00
21 - Eu considero o que meus pares vão dizer quando penso nas possíveis escolhas que eu poderia fazer.	0,86	1,00	0,86	1,00
22 - Se um profissional mais experiente recomenda uma opção em uma situação de tomada de decisão clínica, eu a adoto em vez de procurar outras opções.	0,71	0,86	0,71	0,86
23 - Se um benefício for realmente grande, vou favorecê-lo sem olhar para todos os riscos.	1,00	1,00	1,00	1,00
24 - Eu procuro por novas informações aleatoriamente.	0,86	1,00	1,00	1,00
25 - Minhas experiências anteriores têm pouca relação com a forma como observo ativamente os riscos e benefícios em decisões sobre os pacientes.	0,86	1,00	1,00	0,86
26 -Ao examinar as consequências das opções que posso escolher, estou ciente dos resultados positivos para o meu paciente.	0,85	1,00	1,00	1,00
27 - Eu seleciono opções que obtive sucesso em circunstâncias semelhantes no passado.	0,86	1,00	1,00	1,00
28 - Se os riscos forem sérios o suficiente para causar problemas, descarto a opção.	1,00	1,00	1,00	1,00
29 - Eu escrevo uma lista de consequências positivas e negativas quando estou avaliando uma decisão clínica importante.	0,86	1,00	1,00	0,86
30 - Não peço aos meus pares que sugiram opções para minhas decisões clínicas.	1,00	1,00	0,86	0,86
31 - Meus valores profissionais são inconsistentes com meus valores pessoais.	1,00	1,00	1,00	1,00
32 - Para mim encontrar alternativas parece ser em grande parte uma questão de sorte.	0,67	0,83	0,83	1,00
33 - No ambiente clínico, tenho em mente os objetivos para a rotina do dia.	1,00	1,00	1,00	1,00
34 - Os riscos e benefícios são as coisas mais distantes da minha mente quando tenho que tomar uma decisão.	0,86	1,00	1,00	0,86
35 - Quando tenho que tomar uma decisão clínica, considero as prioridades e padrões institucionais	0,86	0,86	0,86	0,71
36 - Eu envolvo outras pessoas na minha tomada de decisão somente se a situação exigir.	0,86	1,00	1,00	1,00
37 - Em minha busca por opções, incluo até mesmo aquelas que podem ser consideradas “absurdas” ou inviáveis.	1,00	1,00	1,00	0,86
38 - Saber mais sobre os objetivos do paciente faz parte da minha tomada de decisão clínica.	0,86	1,00	1,00	1,00
39 - Examino os riscos e benefícios apenas para consequências que tenham implicações sérias.	1,00	1,00	1,00	0,86
40 - Os valores dos pacientes devem ser consistentes com os meus para que eu possa tomar uma boa decisão.	1,00	1,00	1,00	1,00

*Nota: SEM - semântica; IDIO - idiomática; CULT - cultural; CONC - conceitual.*

Nota-se que todos os componentes e itens do instrumento adaptado atingiram a pontuação de CVR acima de 0,57 na equivalência linguística.

A Tabela 2 mostra o Coeficiente de Validade de Conteúdo da versão preliminar da CDMNS quanto à pertinência, clareza e relevância. Além disso, os itens 6 e 32 foram excluídos, pois não pontuaram o CVR esperado de 0,67 no domínio pertinência. Os itens 2, 19 e 37, com pontuação de 0,67 no domínio de pertinência e clareza, foram revisados e reescritos conforme as sugestões dos juízes.

**Tabela 2:** Coeficiente de Validade de Conteúdo do *Clinical Decision Making in Nursing Scale* da versão preliminar. São Paulo, SP, Brasil, 2022

Escala Preliminar	Pertinência	Clareza	Relevância
ESCALA DE JENKINS PARA TOMADA DE DECISÃO CLÍNICA EM ENFERMAGEM	1,00	1,00	1,00
Instruções: Para cada uma das seguintes afirmações, pense em suas ações ao cuidar de pacientes em ambiente de cuidados intensivos. Responda com base no que você está fazendo agora no contexto clínico.	1,00	0,83	1,00
Não há respostas certas ou erradas. O importante é sua avaliação de como você normalmente age como um tomador de decisões no ambiente clínico. Nenhuma das afirmações abrangem situações de emergência. Circule a resposta que mais se aproxima da maneira como você se comporta. Não é necessário ficar muito tempo em cada resposta.	0,83	1,00	1,00
Responda todos os itens. Isso deve levar aproximadamente dez minutos.	1,00	1,00	1,00
Por favor, utilize a seguinte legenda ao responder a estas perguntas:	1,00	1,00	1,00
Circule de acordo com a forma como você provavelmente se comportaria:	1,00	1,00	1,00
S - Sempre - O que você faz consistentemente todas as vezes	1,00	1,00	1,00
F - Frequentemente - O que você costuma fazer na maioria das vezes	1,00	0,83	1,00
O - Ocasionalmente - O que você faz de vez em quando	1,00	1,00	1,00
R - Raramente - O que faz você raramente	1,00	0,83	1,00
N - Nunca - O que você nunca faz em nenhum momento	1,00	0,83	1,00
Nota: Certifique-se de responder em termos do que está fazendo no ambiente clínico no presente momento	**0,67**	0,83	1,00
1 - Se a decisão clínica for vital e houver tempo, faço uma busca completa por alternativas.	1,00	**0,67**	1,00
2 - Quando uma pessoa está doente, seus valores culturais e crenças são secundários à implementação dos serviços de saúde.	1,00	**0,67**	1,00
3 - Os fatores situacionais do momento determinam o número de opções que exploro antes de tomar uma decisão.	0,83	1,00	1,00
4 - A busca por novas informações ao tomar uma decisão traz mais problemas que compensações.	1,00	1,00	1,00
5 - Eu consulto livros ou literatura profissional para pesquisar coisas que não entendo.	1,00	0,83	1,00
6 - Uma abordagem aleatória para procuraras opções funciona melhor para mim.	**0,50**	**0,50**	1,00
7 - Eu utilizo do método Brainstorming para encontrar ideias pra soluções	1,00	1,00	1,00
8 - Eu me empenho para obter o máximo de informações possível para tomar decisões.	1,00	0,83	1,00
9 - Eu ajudo meus pacientes no exercício de seus direitos de tomar decisões sobre seus próprios cuidados	1,00	1,00	1,00
10 - Quando meus valores estão em conflito com os do cliente, sou objetivo o suficiente para lidar com a tomada de decisão necessária para a situação.	1,00	1,00	1,00
11 - Eu considero os conselhos e opiniões de especialistas, mesmo que essa não seja a escolha que eu faria.	1,00	1,00	1,00
12 - Eu resolvo um problema ou tomo uma decisão sem consultar ninguém, utilizando as informações de que disponho no momento.	1,00	1,00	1,00
13 - Nem sempre paro para examinar todas as possíveis consequências de uma decisão que preciso tomar.	0,83	1,00	1,00
14 - Considero o bem-estar futuro da família quando tomo uma decisão clínica que envolve o paciente.	1,00	1,00	1,00
15 - Tenho pouco tempo ou energia disponível para buscar informações.	1,00	**0,67**	1,00
16 - Eu listo mentalmente as opções antes de tomar uma decisão.	1,00	1,00	1,00
17 - Ao examinar as consequências das opções que posso escolher, geralmente penso “Se eu fizer isso, então ...”.	1,00	1,00	1,00
18 - Eu considero até as consequências mais remotas antes de fazer uma escolha.	1,00	1,00	1,00
19 - O consenso entre meus pares é importante para mim para a tomada de decisão.	1,00	0,83	1,00
20 - Eu incluo os pacientes como fonte de informações	1,00	1,00	1,00
21 - Eu considero o que meus pares vão dizer quando penso nas possíveis escolhas que eu poderia fazer.	1,00	1,00	1,00
22 - Se um profissional mais experiente recomenda uma opção em uma situação de tomada de decisão clínica, eu a adoto em vez de procurar outras opções.	1,00	1,00	1,00
23 - Se um benefício for realmente grande, vou favorecê-lo sem olhar para todos os riscos.	1,00	1,00	1,00
24 - Eu procuro por novas informações aleatoriamente.	1,00	1,00	1,00
25 - Minhas experiências anteriores têm pouca relação com a forma como observo ativamente os riscos e benefícios em decisões sobre os pacientes.	1,00	**0,67**	1,00
26 -Ao examinar as consequências das opções que posso escolher, estou ciente dos resultados positivos para o meu paciente.	1,00	1,00	1,00
27 - Eu seleciono opções que obtive sucesso em circunstâncias semelhantes no passado.	1,00	1,00	1,00
28 - Se os riscos forem sérios o suficiente para causar problemas, descarto a opção.	1,00	1,00	1,00
29 - Eu escrevo uma lista de consequências positivas e negativas quando estou avaliando uma decisão clínica importante.	1,00	**0,67**	1,00
30 - Não peço aos meus pares que sugiram opções para minhas decisões clínicas.	1,00	1,00	1,00
31 - Meus valores profissionais são inconsistentes com meus valores pessoais.	1,00	1,00	1,00
32 - Para mim encontrar alternativas parece ser em grande parte uma questão de sorte.	**0,50**	1,00	1,00
33 - No ambiente clínico, tenho em mente os objetivos para a rotina do dia.	1,00	1,00	1,00
34 - Os riscos e benefícios são as coisas mais distantes da minha mente quando tenho que tomar uma decisão.	1,00	**0,50**	1,00
35 - Quando tenho que tomar uma decisão clínica, considero as prioridades e padrões institucionais	1,00	1,00	1,00
36 - Eu envolvo outras pessoas na minha tomada de decisão somente se a situação exigir.	1,00	1,00	1,00
37 - Em minha busca por opções, incluo até mesmo aquelas que podem ser consideradas “absurdas” ou inviáveis.	1,00	0,83	1,00
38 - Saber mais sobre os objetivos do paciente faz parte da minha tomada de decisão clínica.	1,00	0,83	1,00
39 - Examino os riscos e benefícios apenas para consequências que tenham implicações sérias.	1,00	**0,67**	1,00
40 - Os valores dos pacientes devem ser consistentes com os meus para que eu possa tomar uma boa decisão.	1,00	0,83	1,00

Do ponto de vista técnico da redação dos itens, houve a presença de itens com dois fatores (1, 2, 12, 15, 25, 34, 37 e 39) e a necessidade de adequação dos itens (3,5,9,11, 13, 28, 29, 35). Nota-se que, após os ajustes dessas questões técnicas, surgiu um instrumento com 24 itens com necessidade de modificação, sendo que oitos itens apresentaram dois fatores e 16 itens foram reescritos. Dessa forma, podemos notar que essas modificações configuram uma remontagem (*assembly*)^(16)^.

A Tabela 3 apresenta a análise sobre o instrumento de forma geral, sendo considerado um CVR de 0,67 para cada questão.

**Tabela 3:** Coeficiente de validade do instrumento da versão preliminar do *Clinical Decision Making in Nursing Scale*. São Paulo, SP, Brasil, 2022

Questões sobre o instrumento	CVR
O instrumento mede APENAS o que se propôs?	**0,50**
Os itens atendem ao conceito postulado?	0,67
A linguagem adotada é adequada para a população?	0,67
As dimensões propostas são coerentes com os itens?	**0,45**
A escala é adequada para a mensuração dos itens do instrumento?	**0,37**
O instrumento atende às dimensionalidades propostas?	**0,50**

*Nota: CVR - Content Validity Ratio.*

Nota-se que na escala, dimensionalidade proposta, variável latente estudada, no caso do TDC, o CVR ficou abaixo do esperado.

Na fase de processo de resposta, foi composto por dez enfermeiros com idade média de 28 anos, sendo seis do sexo feminino e com tempo de formação de cinco anos (DP 1,18). Além disso, oito possuem especialização na modalidade residência; sete atuam em Unidade de Terapia Intensiva; e três atuam em unidade de internação. O tempo de trabalho na instituição era de 2,2 anos (DP 1,32), e o tempo de trabalho no setor atual era de 1,7 anos (DP 0,9). Essa etapa foi realizada em duas rodadas. Na primeira rodada, a versão preliminar do instrumento foi aplicada em cinco enfermeiros recrutados da população-alvo. Todos os comentários e sugestões para cada item foram compilados e analisados. Os participantes não relataram dificuldade de leitura do instrumento e instruções de preenchimento. Observou-se que todos os itens foram respondidos, e os itens desmembrados foram dispostos em uma ordem diferente, para evitar que os participantes confundissem durante o preenchimento. Nessa primeira rodada, houve a modificação do item “Escrevo uma lista de consequências, quando estou avaliando uma decisão clínica importante”, pois o ato de escrever foi considerado uma atividade que não se faz, substituindo por “Listo mentalmente as consequências quando estou avaliando uma decisão clínica importante”. E o item 37 foi considerado fora do contexto de atuação, além de colocar o paciente em risco. Diante dos apontamentos, foi realizada uma segunda rodada com cinco enfermeiros; nesta fase, não foram apresentadas dificuldades dos participantes. Observou-se que o tempo de resposta médio foi de dez minutos (tempo mínimo de sete a 13 minutos).

## DISCUSSÃO

A TDC é uma atribuição do enfermeiro que exige conhecimentos técnico-científicos. Um instrumento de TDC irá contribuir para melhorar o currículo das universidades, apoiando as dificuldades dos estudantes de graduação em enfermagem. Os hospitais poderão desenvolver seus profissionais para terem autoeficácia no processo de tomada de decisão no seu ambiente de trabalho e, assim, conseguirem obter melhores desfechos assistenciais.

A escolha de um instrumento de TDC desenvolvido em outra cultura e língua diversa requer a ATC. Esse é um processo complexo que envolve uma sequência de procedimentos metodológicos para garantir que está se medindo a variável latente, apesar de um contexto diferente. Além disso, isso não garante que teremos um instrumento adaptado e validado na nossa cultura e população, mesmo seguindo as recomendações da psicometria contemporânea^(16,17)^.

Nota-se a importância de avaliar a equivalência linguística orientada pelo contexto cultural da variável latente estudada para os enfermeiros brasileiros. Nessa perspectiva, chama a atenção o contexto cultural da competência de TDC pelos enfermeiros brasileiros no cenário atual, uma vez que o instrumento foi desenvolvido na década de 1980, no contexto histórico e cultural dos enfermeiros americanos. O CDMNS é o instrumento mais utilizado nas publicações científicas em vários países do mundo no que se refere à investigação de TDC.

No presente estudo, utilizaram-se dois referenciais metodológicos com as recomendações do protocolo FACT ^(14)^, alinhados com o referencial de Beaton *et al*.^(18)^, fornecendo maior robustez nesse processo. Não houve discrepâncias significativas nas versões apresentadas pelos tradutores, sendo que aquelas que surgiram foram solucionadas pelos pesquisadores. Observou-se que realizar a equivalência não é o suficiente, pois replicaria os possíveis erros do instrumento; por isso, é necessário realizar o painel de juízes para verificar outras questões relacionadas ao conteúdo e ao instrumento de uma forma geral^(16)^.

No entanto, apenas no trabalho de Kouravand^(19)^, que adaptou e validou o CDMNS para a cultura persa, adotou-se o protocolo de Beaton para o sequenciamento da ATC. Outros artigos não citaram protocolos norteadores, contribuindo para a fragilidade^(20-22)^.

Dentro do processo de ATC, na versão brasileira, não foi realizada a retrotradução, devido ao domínio público do instrumento após o falecimento da autora. Essa etapa tem sido relatada por Epstein *et al*.^(23)^ como tendo impacto moderado. O comitê de juízes ajuda, por sua vez, a garantir um conteúdo preciso, considerando que sejam bilingues e conhecedores da variável latente no contexto a ser adaptado. Uma das principais funções do comitê é garantir que o conteúdo do questionário permaneça verdadeiro ao original.

Outro ponto importante foi a adoção do CVR aplicado para as análises das respostas dos juízes, utilizando a pontuação dicotômica, que exige o posicionamento sobre suas escolhas; este índice apresenta superioridade de Índice de Validade de Conteúdo (IVC) e permite a inclusão de um número maior de juízes^(24,25)^. Isso ocorre ao contrário da recomendação do IVC de Polit^(26)^ que usualmente pode gerar viés, visto que o índice não é ajustado ao número de juízes como no CVR crítico. Porém, as recomendações de Epstein *et al*.^(23)^ especificam que o painel de juízes deve ser composto por pesquisadores, tradutores e profissionais, metodológicos e leigos. Tendo em conta essas recomendações mais recentes, seria arriscado restringir as opiniões a cinco especialistas, o que poderia enviesar as análises.

Dos resultados obtidos, este estudo destoou das publicações de validação do CDMNS em outros países^(20-22)^, uma vez que a tradução adaptada apresenta questões técnicas da redação do instrumento pontuadas pelo comitê de juízes, tais como itens com dois fatores, fragilidade no formato de escala e sugestões de reformulação da redação. Tais resultados permitem dizer que o processo de ATC se enquadra como uma remontagem, devido à reformulação do conteúdo adaptado divergir do original^(16)^.

Diante do exposto, Coons^(27)^ e Steward^(28)^ afirmam que o rigor da equivalência deveria variar de acordo com a magnitude das modificações. Todavia, independentemente do nível de modificações, recomenda-se testar as modificações por meio de entrevista cognitiva, teste de usabilidade e medidas psicométricas^(25)^.

No cerne das alterações do formato de resposta do instrumento, no estudo de Minish da Palestina^(22)^, o comitê de juízes sugeriu o uso de uma escala do tipo Likert de 3 pontos (1 = nunca; 2; frequente; 3 ocasionalmente). Já o estudo de Girot^(29)^ utiliza uma escala tipo Likert de 5 pontos de concordância, conforme a primeira publicação sobre o desenvolvimento do instrumento^(10)^. Para testar essas modificações, sugerem-se teste de usabilidade e propriedades psicométricas em geral.

Nesse contexto, a elaboração de instrumento, mesmo que adaptado, requer uma boa redação e um formato de escala adequado para mensurar a variável latente, que minimizará o impacto da estrutura interna e proporcionará interpretação dos resultados tanto para pesquisadores quanto para os usuários do instrumento^(17)^.

Nos estudos de validação do CDMNS de Duarte^(30)^ e Kouravand^(19)^, houve a exclusão de 17 itens do instrumento original na fase da estrutura interna. Neste estudo, notou-se que, na fase de conteúdo do teste, alguns dos itens mencionados já haviam sido excluídos. Destacou-se a relevância da característica do painel de juízes multidisciplinar, com experiência em estudos psicométricos, que, além de analisar o conteúdo do instrumento, também verifica outros aspectos relacionados à redação do item, ao formato de resposta e à compreensão dos itens na etapa de processo de resposta.

Observou-se a necessidade da remontagem do CDMNS na versão brasileira. Segundo Iliescu, a remontagem de um instrumento preocupa-se com uma nova medida na cultura alvo, onde foi gerado um novo conteúdo. Diante do resultado das modificações dos itens, foi gerado um novo conteúdo que requer uma reflexão sobre a TDC no contexto brasileiro e o dilema da autopercepção dos enfermeiros na sua prática, que pode ser uma opção a ser desenvolvida para fornecer à cultura brasileira uma medida funcional, útil e válida do construto alvo.

Outro ponto importante sobre a importação do instrumento para cultura brasileira como mencionado anteriormente se refere à questão de ser tratar de país continental com as suas diferenças socioeconômico-culturais e modelos de gestão hospitalares que podem ser considerados como fatores contextuais importantes ao considerar o contexto cultural do instrumento original.

Nota-se que os artigos de validação do CDMNS publicados^(20-22)^ se referem ao processo de resposta como pré-teste, não dando a sua nomenclatura correta, e pouco é relatado sobre como são realizadas as técnicas, ou seja, cita-se como o pré-teste foi realizado, embora não contabilizando o tempo de resposta^(20-22)^. No artigo original^(10)^, foi citado o tempo de resposta semelhante ao apresentado neste estudo, que foi de 12 minutos.

### Limitações do estudo

Este estudo possui algumas limitações. O CDMNS, traduzido para a língua portuguesa do Brasil, é um material desenvolvido em décadas anteriores que, hoje, pode ter um impacto totalmente diferenciado quando aplicado ao contexto, visto as mudanças geracionais dos enfermeiros e a diversidade das características dos hospitais no cenário do nosso país continental. No processo de adaptação, deve-se garantir que o conceito do instrumento é igual ao original não apenas para a língua, mas também para o tempo e o contexto. É possível considerar as barreiras ao fazer uma comparação direta entre nações, culturas e tempo. Houve necessidade de ter dois painéis de juízes para avaliar a equivalência e outro painel de juízes para avaliar o conteúdo que abrange as regiões do nosso país continental.

### Contribuições para as áreas da saúde, enfermagem ou políticas públicas

Os achados deste estudo contribuem com o avanço do conhecimento na área da enfermagem, colaborando para melhorar as fragilidades encontradas no que tange à TDC a beira leito, tanto para os estudantes quanto para os enfermeiros. Nota-se a importância de outros estudos com referenciais contemporâneos que norteiam o processo de remontagem do instrumento para o contexto brasileiro, dando continuidade ao processo das evidências de validade de um instrumento de TDC para os enfermeiros em nosso país.

## CONCLUSÕES

O CDMNS foi traduzido e adaptado culturalmente para o Brasil de acordo com a metodologia preconizada pela literatura. A versão brasileira representa uma remontagem do instrumento original, demonstrando equivalência linguística, mas precisará ser testado na estrutura interna para verificar a interpretação dos itens. A evidência de conteúdo do teste apresenta fragilidade do ponto de vista técnico, com necessidade de testes para a sua validação. O processo de resposta apresenta boas evidências de validade na interpretação do conteúdo dos itens pelos participantes. Este instrumento servirá como uma estratégia para fornecer subsídios às universidades e organizações, para promover estratégias nos currículos de enfermagem e contribuir para o desenvolvimento contínuo nas organizações para apoiar a TDC do enfermeiro no seu ambiente de trabalho.

## Data Availability

Os dados de pesquisa estão disponíveis no corpo do artigo.
